# Exploration of the Educational Utility of National Film Using Deep Learning From the Positive Psychology Perspective

**DOI:** 10.3389/fpsyg.2022.804447

**Published:** 2022-06-09

**Authors:** Yangzhen Zhaxi, Yueting Xiang, Jilin Zou, Fengrui Zhang

**Affiliations:** ^1^School of Art, Southwest Minzu University, Chengdu, China; ^2^School of Foreign Languages and Cultures, Xichang University, Xichang, China; ^3^Department of Education, Linyi University, Linyi, China; ^4^College of Life Science, Sichuan Agricultural University, Ya'an, China

**Keywords:** psychology, deep learning, national films, educational function, convolutional neural network

## Abstract

The research focuses on the application of positive psychology theory, and studies the educational utility of national films by using deep learning (DL) algorithm. As an art form leading China's film and TV industry, national films have attracted the interest of many domestic scholars. Meanwhile, researchers have employed various science and technologies to conduct in-depth research on national films to improve film artistic levels and EDU-UTL. Accordingly, this paper comprehensively studies the EDU-UTL of national films using quality learning (Q-Learning) combined with DL algorithms and educational psychology. Then, a deep Q-Learning psychological model is proposed based on the convolutional neural network (CNN). Specifically, the CNN uses the H-hop matrix to represent each node, and each hop indicates the neighborhood information. The experiment demonstrates that CNN has a good effect on local feature acquisition, and the representation ability of the obtained nodes is also powerful. When *K* = 300, the psychological factor Recall of Probability Matrix Decomposition Factorization, Collaborative DL, Stack Denoising Automatic Encoder, and CNN-based deep Q-Learning algorithm is 0.35, 0.71, 0.76, and 0.78, respectively. The results suggest that CNN-based deep Q-Learning psychological model can enhance the EDU-UTL of national films and improve the efficiency of film education from the Positive Psychology perspective.

## Introduction

Today, the world economy is flourishing under long-term scientific and technological development, and the global pattern is changing faster than ever. Living in such a transitional era, people increasingly pursue spiritual wellbeing with their overall quality improved. In particular, the film and TV works' educational utility (EDU-UTL) (Bajorek and Gawroński, 2018; Sung et al., 2019; Mansyur and Suherman, 2020) is getting the attention of a proliferation of researchers and educators. It is universally acknowledged that change in the world is inseparable from the promotion of modern civilization, and the film is just an intuitive way to illustrate human achievements and the civilizational process. The modern film industry is not only a medium, but also plays a vital role in people's daily lives. For example, recent years have witnessed the increasing influence of national films (Carroll Harris, 2018; Hoyler and Watson, 2019; Kim, 2019) on the film market regarding their EDU-UTL (Andersson, 2019; Traverso et al., 2019; Paoletti et al., 2020). As a result, national films' market share is rising and dominating, including *Operation Red Sea, War Wolf 2*, and *Changjin Lake*. These films reproduce the gunfire scenes during the war with modern technologies and bring the audience a tremendous spiritual impact, thus making people cherish the peace and prosperity bought at the cost of their predecessors' lives. In addition, national films continually educate the mass to remember the historical meaning of the hard struggle and maintain the unyielding determination and tenacious will in the face of difficulties.

Both Chinese and international researchers have done much research on the EDU-UTL of national films from the perspective of Positive Psychology. Rogoza et al. (2018) argued a positive correlation between people's fragile narcissistic behavior and admiration psychology. They proved the importance of environmental influence on personality traits from a deeper Positive Psychology perspective. Thus, the EDU-UTL of national films was undoubtedly an excellent alternative to remove adverse effects and instill positive elements. Chen (Chen, 2019) said that, from a psychological point of view, ones' characteristics would affect their work performance. In addition, different experiences in job transfers and external environmental changes might often impact their life, work, and other social behaviors. Then, the EDU-UTL of national films was combined with personal growth to help individuals deal with strange real-life scenarios and give them spiritual guidance. Wu and Song (2019) proposed the importance of trust as a psychological element to an entrepreneurial group from the perspective of entrepreneurship. They suggested that faith was essential for interpersonal relationships in daily life; in addition, a mutual trust could uplift work efficiency and the quality of life. Trust cultivation could also be learned from some excellent films, apart from interpersonal communication.

According to relevant research, this paper investigates the EDU-UTL of national films and novel research methods. Specifically, this paper uses the deep learning (DL) algorithm to study the EDU-UTL of national films from the Positive Psychology perspective. The main innovation lies in the combination of the DL algorithm and Positive Psychology. So far, there are few studies on the fusion of the DL algorithm and relevant knowledge in psychology. The research findings have laid an essential theoretical foundation for the national film's follow-up study and have a paramount reference significance for relevant personnel engaged in researching the EDU-UTL of national films.

## Literature Review

Many scholars have done extensive research on the EDU-UTL of national films. For example, Orlandi et al. (2018) studied University education and inter-race relations from an outlook of otherness relations between universities and external communities. They found that cultural intervention through films and other means could promote the initial training of graduates and enhance the EDU-UTL of national films. Williams et al. (2019) educated patients with stroke on stroke drug preparation through stroke treatment drugs in movies. Research showed that films had EDU-UTL and could intervene in people's educational level. Smits and Janssenswillen (2020) transferred the theoretical principles to different classroom environments through a reasonable curriculum plan based on language teachers' cross-case exploration and research on racial diversity methods. The results corroborated that appropriate teaching practice could produce a perfect sense of professional diversity. Scholar Jin (2020) studied the translation of the Chinese national film language from a historical perspective. He investigated the translation policies, institutions, and models of minority languages in China with official regulations, newspapers, quantitative data, memoirs, and oral history. The study revealed the changing trend of the Chinese national film language over time. To sum up, exploring the EDU-UTL of national films from the perspective of psychology is conducive to improving the impact of national films on educational psychology and significantly affecting people's mental health. Therefore, this paper studies the structure of DL and AlexNet Neural Networks using the psychological model based on quality learning (Q-Learning). After the deep separable model's training, the Q-Learning model analyzes the EDU-UTL of films in different data sets, and film recognition accuracy and Recall are analyzed based on the DL model. This research has a practical reference value to apply the Q-Learning network in the field of national film education.

## Research Model and Framework

### Psychological Model for Q-Learning

**Figure 1:**
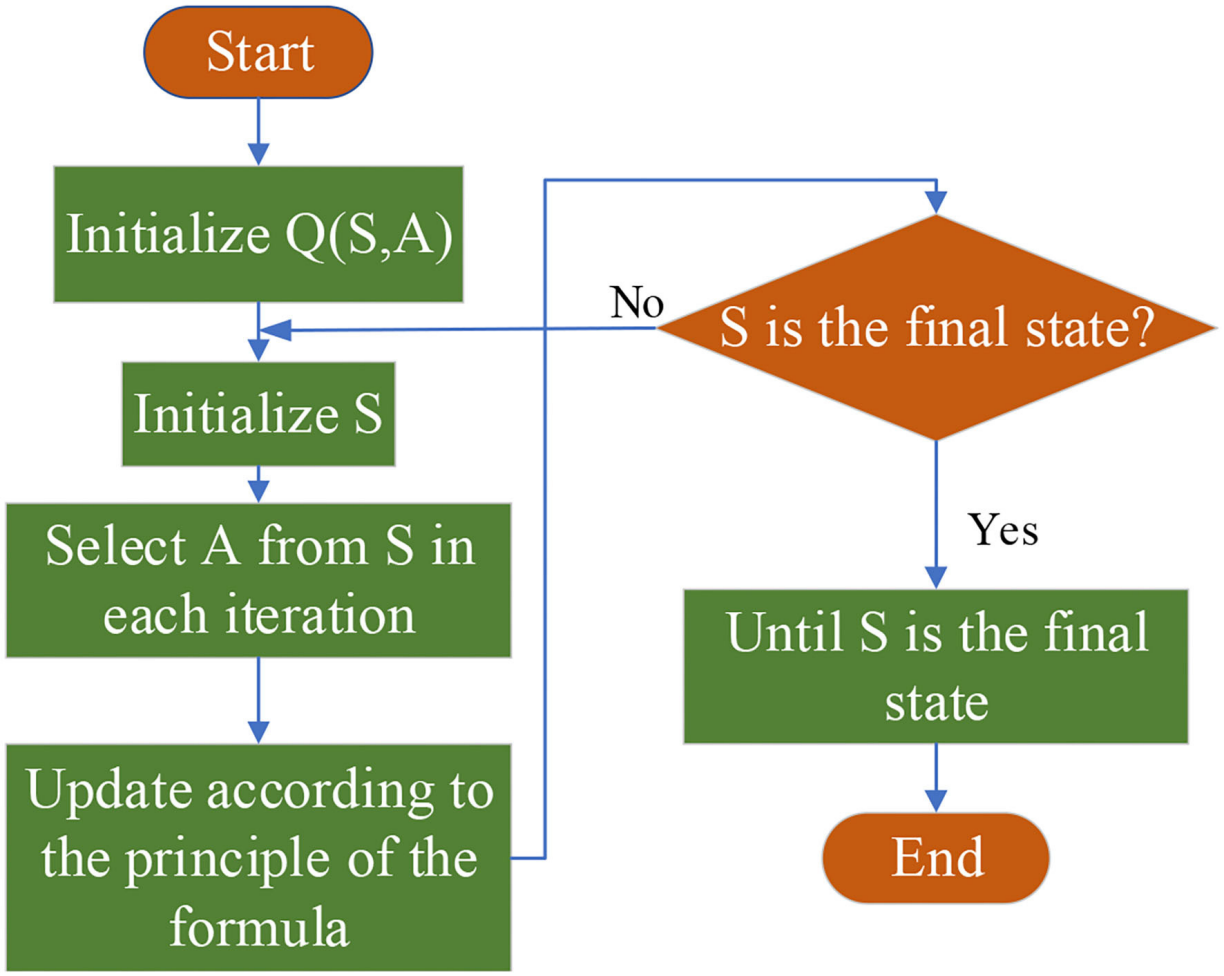
Quality learning (Q-Learning) algorithm framework.

Reinforcement learning (RL) is a machine learning (ML) algorithm to solve the network reinforcement function. Its ultimate goal is to achieve the maximum expected evaluation according to the network parameters. Therefore, this paper proposes an active RL method, namely, Q-Learning. The core of the Q-Learning method is the optimization strategy, including state, action, and reward. The process of the Q-Learning algorithm (Zhang et al., 2018; Jang et al., 2019; Low et al., 2019) reads: firstly, *Q*(*S, A*) is initialized, and the state set *S* is initialized in every iteration; secondly, *Q* strategy is implemented, and action *A* is selected at state *S* in each iteration; iteratively, *Q*(*S, A*) is updated until *S* reaches the final state. Figure 1 shows the specific algorithm framework of Q-Learning.

### DL Model

This section elaborates on the DL model, such as the popular recurrent neural network (RNN) (Bueno et al., 2018; Müller and Hiss, 2018; Pang et al., 2020). The sequence segmentation method is employed for psychological factors because of its non-overlapping feature. Therefore, the sequence label is introduced to transform the sequence segmentation into sequence labeling by labeling each psychological factor and the word in the segmentation (label unit). Then, the Intermediate Other Begin-2 (IOB2) tag set is chosen, in which the letter *B* represents the starting word in the label unit, the letter *I* means that the word belongs to the middle word or ending word. *O* denotes that the word does not belong to any label unit. Finally, the sequence label set has transformed the psychological factors into a sequence of brands through the following steps. Firstly, the goal and framework of psychological factors are given, and each psychological factor is appropriately labeled as *T* using the sequence tag set IOB2 to generate a tag sequence. Furthermore, the label sequence of psychological factors is optimized using Equation (1).


(1) $$\begin{array}{l} T^{*}=\arg\max P\left(T=t_{1},t_{2},\cdots \;,t_{n}|S=w_{1},w_{2},\cdots \;,w_{n},f_{w_{t}}\right) \end{array}$$

In Equation (1), T* refers to a proper sequence that can recover the information about the psychological factors. Traditionally, a statistical ML algorithm is chosen to solve the conditional probability of psychological factor optimization presented in Equation (1). Usually, these algorithms first extract the features from the psychological factors; then, the model learns through a training data set to determine the best feature combinations and hyperparameters used for data verification on the testing data set. However, these algorithms also have some shortcomings, such as excessive dependence on manual feature construction and high cost. Fortunately, with the wide application of DL algorithms (Han et al., 2018; Sirignano and Spiliopoulos, 2018; Wang et al., 2018), neural network methods have achieved remarkable results in labeling tasks of psychological factors. In particular, as an improved RNN, the Bi-directional long short-term memory (BiLSTM) neural network has become the mainstream algorithm in the DL field. Based on the above, psychological factor feature extraction is transformed into a sequence labeling task. Now that RNN can process sequence data, it is applied to psychological factor sequence labeling. Specifically, the label sequence is input through RNN model, and then the model is reproduced along the evolution direction of the sequence, and all network nodes are linked. The recurrent equation of standard RNN reads:


(2) $$\begin{array}{l} h_{t}=H\left(W\left[h_{t-1},x_{t}\right]+b\right) \end{array}$$

In Equation (2), *h*_*t*_ refers to the hidden layer output; *H* represents a nonlinear function, such as a simple TanH function or a series of very complex transformations; *W* represents the weight. In the RNN, the hidden layer input at time *t* includes the current input *x*_*t*_ and the hidden layer output *h*__*t*_ − 1_ from the last time. Therefore, other psychological factors can be referred to predict the *t*th psychological factor. Yet, the standard RNN has two deficiencies. On one hand, the long distance dependence of the sequence is difficult to capture for RNNs under excessively long gradient transition time. On the other hand, gradient disappearance or gradient explosion occurs when dealing with long sequences. long short-term memory (LSTM) is the most commonly optimized network based on the standard RNN (Fischer and Krauss, 2018; Hu et al., 2018; Kratzert et al., 2018). The LSTM network has three additional gate structures in the hidden layer to memorize, update, and utilize information: input, forget, and output gates. These processes are realized by the Sigmoid function, as shown in Equation (3).


(3) $$\begin{array}{l} f_{t}=\sigma \left(W_{f}\cdot \left[h_{t-1},x_{t}\right]+b_{f}\right) \end{array}$$

In Equation (3), *W*_*f*_ means the weight matrix, *x*_*t*_ denotes the current input, *h*_*t*_ describes the output of the previous step, and *b* represents the offset. Additionally, *i* determines which input information needs to be stored in the cell state. The specific description is shown in Equation (4).


(4) $$\begin{array}{l} i_{t}=\sigma \left(W_{i}\cdot \left[h_{t-1},x_{t}\right]+b_{i}\right) \end{array}$$

In Equation (4), *W*_*i*_ denotes the weight matrix of *i*. The state gate decides how to update the unit state according to *i* and adds the unit state to the information controlled by *f* through *C*_*t*_ to generate new memories.


(5) $$\begin{array}{l} {\tilde{C}}_{t}=\tanh\left(W_{C}\cdot \left[h_{t-1},x_{t}\right]+b_{C}\right) \end{array}$$

(6) $$\begin{array}{l} C_{t}={f_{t}}^{*}C_{t-1}+{i_{t}}^{*}{\tilde{C}}_{t} \end{array}$$

In Equation (5), *W*_*C*_ means the weight matrix generated by a unit state. Finally, the output information is determined. The determined part *h*_*t*_ is selectively output to the new memory:


(7) $$\begin{array}{l} o_{t}=\sigma \left(W_{o}\cdot \left[h_{t-1},x_{t}\right]+b_{o}\right) \end{array}$$

(8) $$\begin{array}{l} h_{t}={o_{t}}^{*}\tanh\left(C_{t}\right) \end{array}$$

In Equations (7) and (8), *W*_*o*_ represents the weight matrix (Ermagun and Levinson, 2019a,b; Gunasekaran and Joo, 2019). Although the LSTM network is based on the standard RNN, the standard RNN or LSTM can only capture the historical sequential information. By comparison, BiLSTM uses two LSTMs in the hidden layer to model the sequence forward and backward and connect their outputs. The backward RNN is shown in Equation (9).


(9) $$\begin{array}{l} h_{t}^{l}=H\left(W^{l}\cdot \left[h_{t-1}^{l},x_{t}\right]+b^{l}\right) \end{array}$$

The forward RNN can be described as given in Equation (10).


(10) $$\begin{array}{l} h_{t}^{r}=H\left(W^{r}\cdot \left[h_{t-1}^{r},x_{t}\right]+b^{r}\right)h_{t}=\left[h_{t}^{l}:h_{t}^{r}\right] \end{array}$$

In Equation (10), *h*_*t*_ = [$h_{t}^{l}$: $h_{t}^{r}$] means that at time *t*, two vectors $h_{t}^{l}$ and $h_{t}^{r}$ are spliced together from beginning to end. BiLSTM replaces the cycle unit in the bidirectional RNN (BiRNN) with an LSTM structure, fusing the advantages of BiRNN and LSTM.

**Figure 2:**
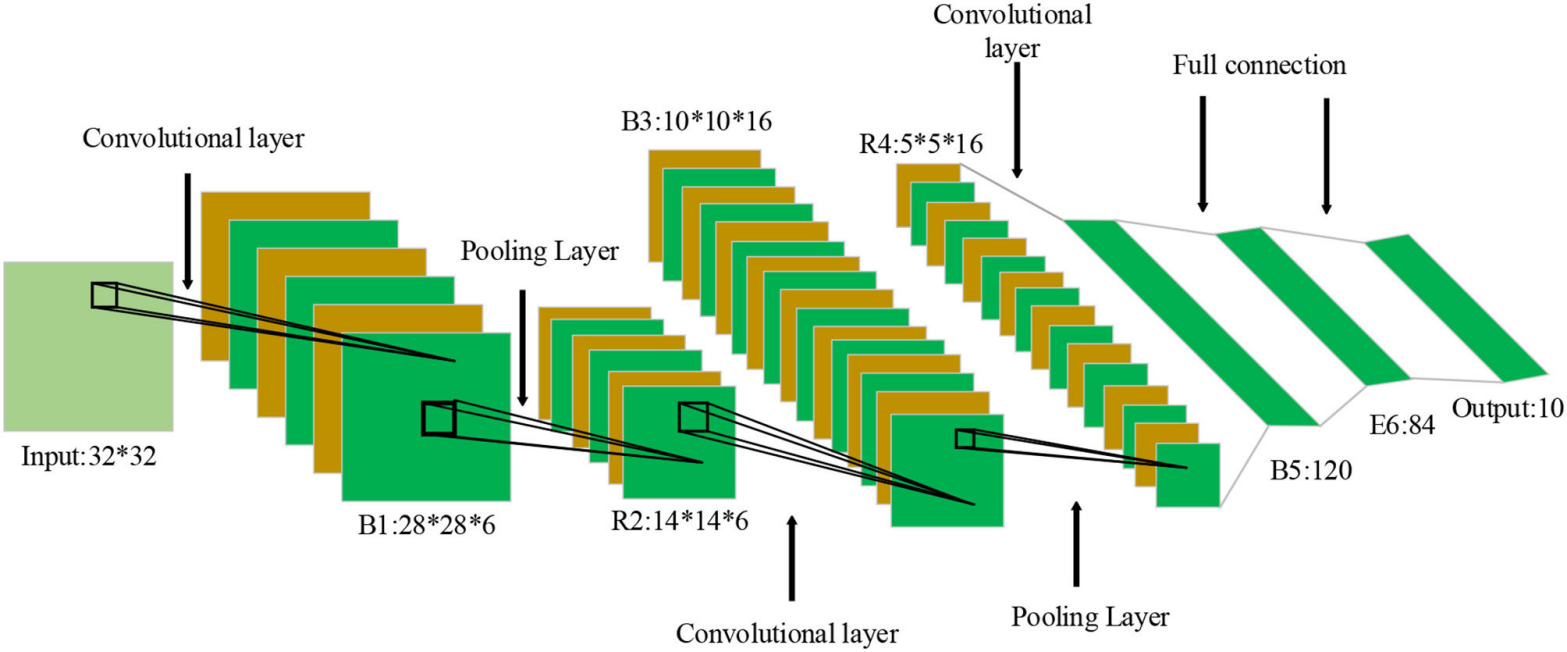
Convolutional neural network (CNN) structure. *Indicates the size of different connection layers of neural network.

The deep convolutional neural network (DCNN) is a typical neural network structure widely used in Computer Vision fields, such as image recognition, face recognition, and target location. Local perception and weight sharing of the convolution layer reduce the number of parameters and the complexity of the network structure; pooling streamlines the feature map and model parameters. Convolutional neural networks (CNNs) can solve the gradient vanishing and the frequent overfitting phenomenon in complex networks with the Sigmoid Activation Function. DCNN-based learning algorithm still has shortcomings. Thus, this section proposes an efficient deep hash learning model based on semantic retention. Given that BiLSTM cannot pay attention to local features, the CNN (Perol et al., 2018; Ting et al., 2019; Dhillon and Verma, 2020) algorithm is introduced to analyze the role of psychological factors from the perspective of Positive Psychology. Figure 2 illustrates a common CNN.

The experimental data are collected and processed by the CNN algorithm. CNN is a feedforward algorithm with the best input and performance. In a CNN, neurons are organized to facilitate data processing and screening. The model parameters include the input, kernel function, and output (also called feature map). Usually, CNN performs convolution operations on multiple dimensions. When a two-dimensional (2D) matrix I is input, the 2D convolution kernel satisfies Equation (11).


(11) $$\begin{array}{l} S\left(i,j\right)=\left(I\cdot K\right)\left(i,j\right)=\underset{m}{\sum }\underset{n}{\sum }I\left(m,n\right)K\left(i-m,j-n\right) \end{array}$$

In Equation (11), *i, j, m*, and *n* are fixed parameters representing the matrix's dimension and order. Convolution can be exchanged and written equivalently as Equation (12).


(12) $$\begin{array}{l} S\left(i,j\right)=\left(I\cdot K\right)\left(i,j\right)=\underset{m}{\sum }\underset{n}{\sum }I\left(i-m,j-n\right)K\left(m,n\right) \end{array}$$

The flipped convolution kernel provides an interchangeable feature for convolution operation: the input index decreases with the kernel index. Kernel flipping can achieve interchangeability, which helps verification tasks, but it is unimportant for neural networks. Typically, NNs can be implemented with the Cross-Correlation function, which is almost the same as the convolution operation but cannot flip the kernel:


(13) $$\begin{array}{l} S\left(i,j\right)=\left(I\cdot K\right)\left(i,j\right)=\underset{m}{\sum }\underset{n}{\sum }I\left(i+m,j+n\right)K\left(m,n\right) \end{array}$$

**Figure 3:**
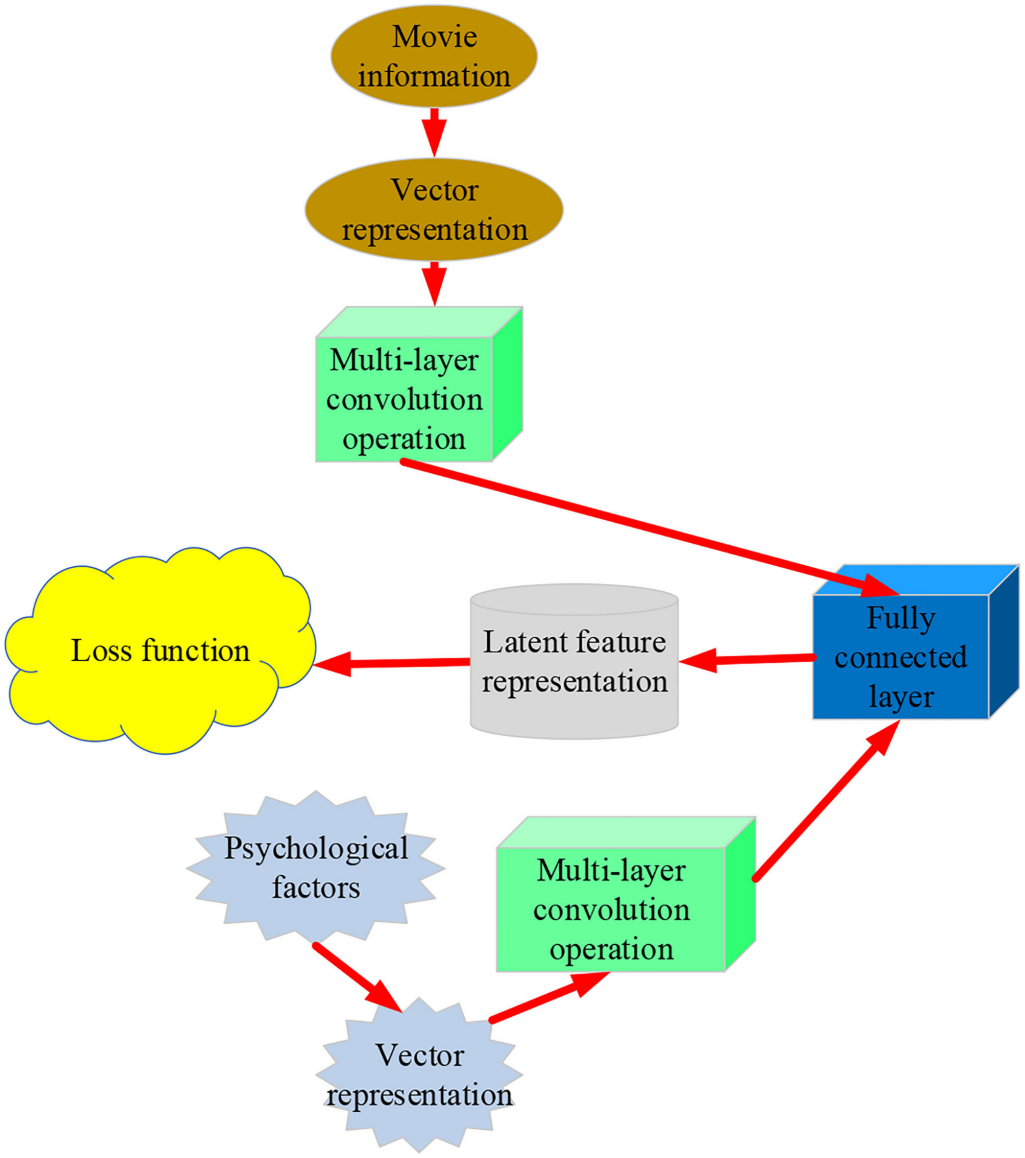
Framework of the CNN.

Any neural network that uses matrix multiplication, but does not depend on the unique properties of the matrix structure, is suitable for convolution operation without significant modification. CNN keeps adjusting its learning rate γ to control the update of weight *w* and deviation *b*. It can identify the closest correct psychological factors by minimizing the loss function. Usually, CNN uses sparse interaction and parameter sharing to improve the DL feature extraction system to improve the efficiency of processing large-scale data input. Figure 3 displays a CNN frame.

**Figure 4:**
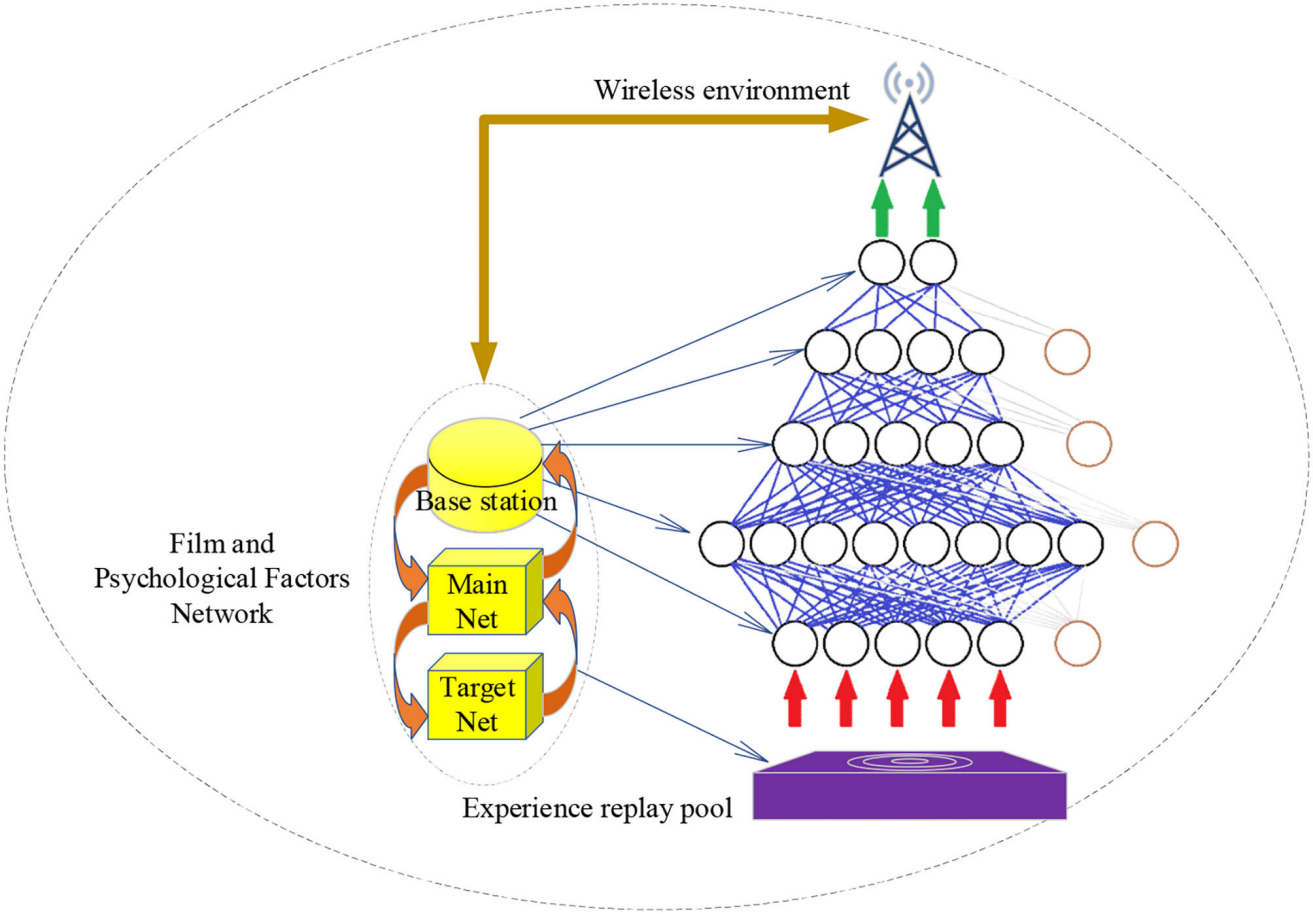
Application architecture of CNN.

AlexNet is a DCNN algorithm with many network layers and strong learning ability. The experiment reported here selects the AlexNet to reduce computation and enhance the network generalization ability. The model parameters are set as follows: the iteration number is 140, the learning rate is 0.002, the batch size is 128, and the convolution kernel's size is 1 × 3. Then, the model uses rectified linear unit (ReLU) Activation Function with the Dropout Rate of 0.5 and the Adam optimizer. The application architecture of CNN is given in Figure 4.

AlexNet's convolution layer has been dramatically improved, bringing robust generalization ability for the network, minimizing overfitting, and shortening the training time. Moreover, overlapping pooling before local normalization can preserve data and eliminate redundancy as much as possible.

The *t*th feature map $y_{t}^{l}\left(i,j\right)$ of the *l*th convolution layer is sampled using overlapping pooling, as presented in Equation (14).


(14) $$\begin{array}{l} a_{t}^{l}(i,j)=\max\{y_{t}^{l}(i,j),\;is\leq i\leq is+w_{c}-1,\\ \;js\leq j\leq js+w_{c}-1\} \end{array}$$

In Equation (14), *s* signifies the pooling step, and *w*_*c*_ indicates the width of the pooling area, *w*_*c*_ > *s*. A local normalization layer is added after the first and second pooling layers of AlexNet to normalize the feature map $c_{t}^{l}\left(i,j\right)$:


(15) $$\begin{array}{l} c_{t}^{l}\left(i,j\right)=a_{t}^{l}\left(i,j\right)/{\left(k+\alpha \overset{\min\left(N-1,t+m/2\right)}{\underset{\max\left(0,t-m/2\right)}{\sum }}{\left(a_{t}^{l}\left(i,j\right)\right)}^{2}\right)}^{\beta } \end{array}$$

The values of the hyperparameters are 2, 0.78, 10^−4^, and 7, respectively, and *N* is the total number of convolution kernels in the first convolution layer. ReLU is used to activate the convolution output to prevent gradient dispersion:


(16) $$\begin{array}{l} y_{t}^{l}\left(i,j\right)=f\left(S_{t}^{l}\left(i,j\right)\right)=\max\left\{0,S_{t}^{l}\left(i,j\right)\right\} \end{array}$$

where *f* (·) represents ReLU. Then, the Dropout Rate is set to 0.4 to avoid overfitting in the fully connected layer. In Equation (16), when *l* = 5, the feature maps can reconstruct a high-dimensional single-layer neuron structure *C5*. Then, the input $Z_{i}^{6}$ of the *i*th neuron in the sixth fully connected layer reads:


(17) $$\begin{array}{l} Z_{i}^{6}=W_{i}^{6}C^{5}+b_{i}^{6} \end{array}$$

where $W_{i}^{6}$ and $b_{i}^{6}$, respectively, refer to the weight and offset of the *i*th neuron in the sixth fully connected layer.

The model generalization improvement process abandons the neuron's output in the sixth and seventh fully connected layers, and $r_{j}^{l}\tilde{\;}bernoulli\left(dp\right),{\tilde{C}}^{l}=r^{l}C^{l}$. Then, the input $Z_{i}^{l+1}$ of the *i*th neuron in the seventh and eighth fully connected layers is $W_{i}^{l+1}{\tilde{C}}^{l}+b_{i}^{l+1}$, and the output $C_{i}^{l}$ of the *i*th neuron in the sixth and seventh fully connected layers is $f\left(Z_{i}^{l}\right)$, namely, $\max\left\{0,Z_{i}^{l}\right\}$. Finally, the input *q*^*i*^ of the *i*th neuron in the eighth fully connected layer can be obtained according to Equation (18).


(18) $$\begin{array}{l} q^{i}=soft\max\left(\overset{8}{\underset{i}{Z}}\right)=\frac{e^{Z_{i}^{8}}}{\sum_{j=1}^{12}e^{Z_{i}^{8}}} \end{array}$$

Meanwhile, the cross-entropy loss function for the classification is used as the model error function:


(19) $$\begin{array}{l} Loss=\sum_{i=1}^{K}y_{i}\cdot \log\left(p_{i}\right) \end{array}$$

(20) $$\begin{array}{l} p_{i}=\frac{\exp\left({ỹ}_{i}\right)}{\sum_{i=1}^{K}\exp\left({ỹ}_{j}\right)} \end{array}$$

where *K* refers to the number of categories, *y*_*i*_ represents the real category distribution of samples, ỹ_*i*_ denotes the output result of the neural network, and *p*_*i*_ stands for the classification result of the Softmax classifier.

The input of the Softmax function is an *N*-dimensional real vector, which is set as *x*, and the calculation reads:


(21) $$\begin{array}{l} \xi {\left(x\right)}_{i}=\frac{e^{x_{i}}}{\overset{N}{\underset{n=1}{\sum }}e^{x_{i}}},i=1,2,...,N \end{array}$$

**Figure 5:**
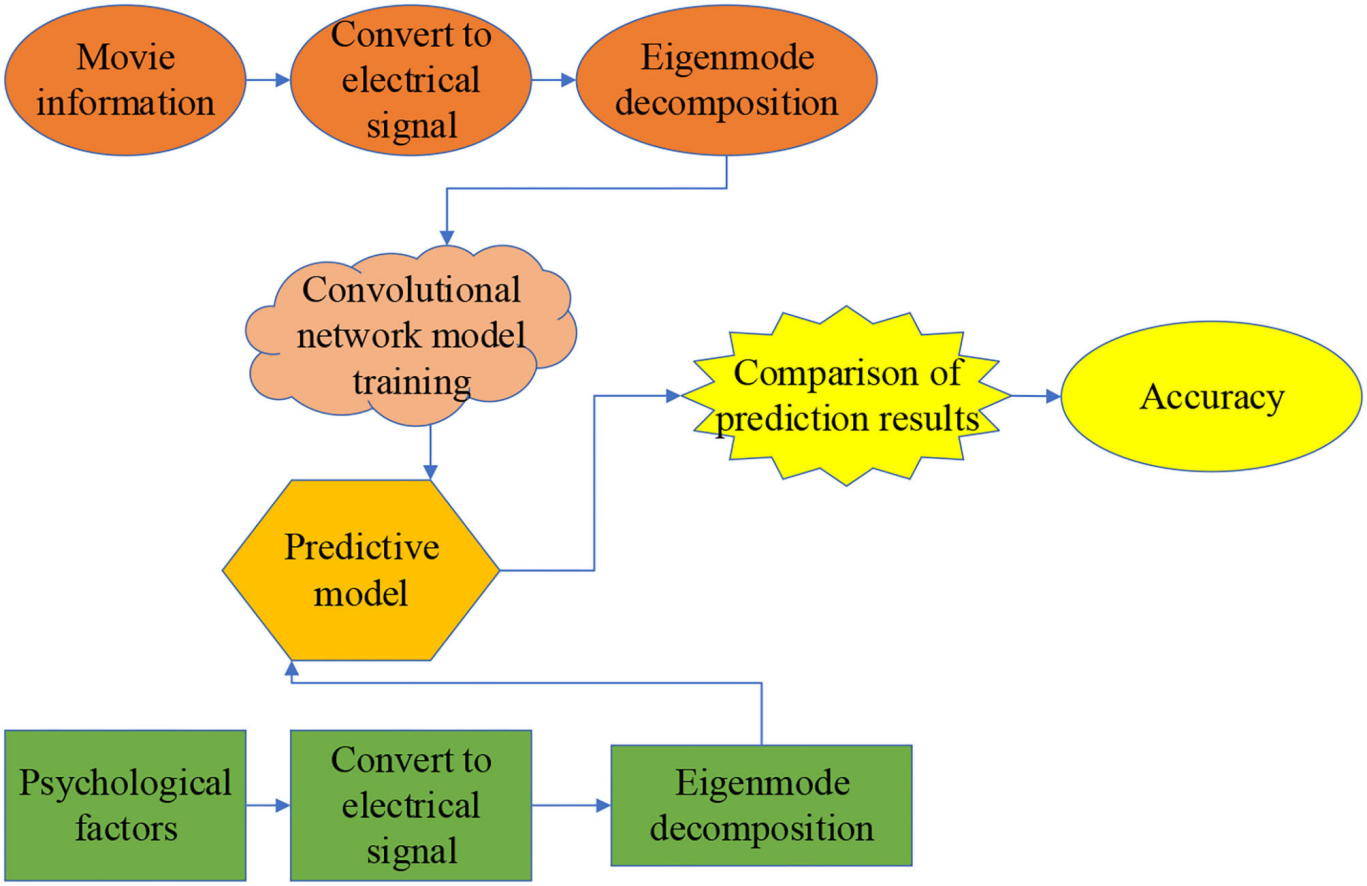
Training model of the separable deep convolutional neural network (DCNN).

The Softmax function can map an *N*-dimensional arbitrary real number vector into an *N*-dimensional vector with all elements normalized between (0, 1). Figure 5 demonstrates the training model of a separable DCNN.

## Experimental Design and Performance Evaluation

### Analysis of Film Education Based on Q-Learning

**Figure 6:**
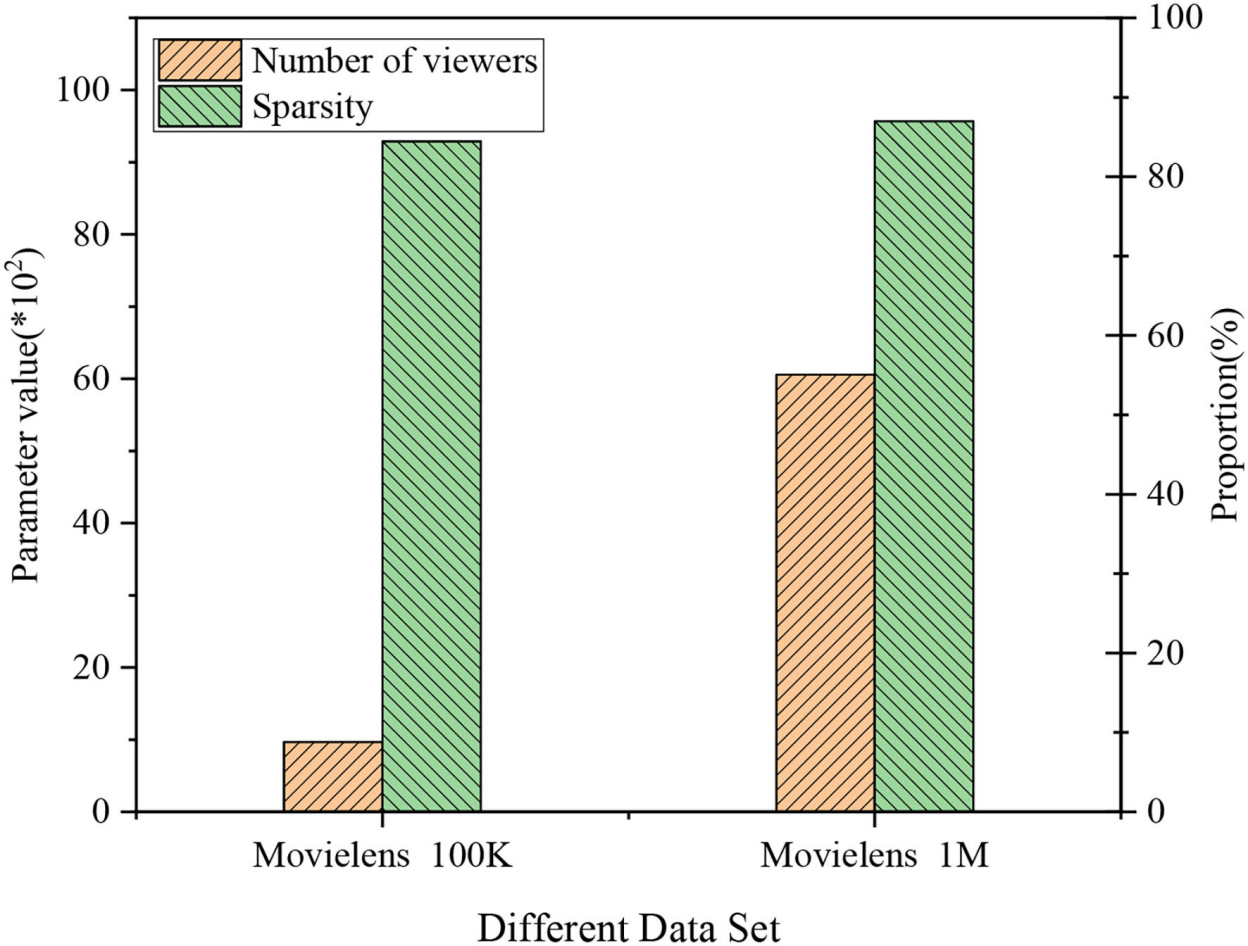
Data histogram of different data sets. *Indicates the size of the parameter value multiplied by 100.

This section explains the EDU-UTL of national films based on DL from an outlook of Positive Psychology. As in Figure 6, the training data set based on Movielens 100K contains 968 movie viewers, with a sparsity of 92.9%; the testing data set based on Movielens 1M contains 6,058 movie viewers, with a sparsity of 95.68%. The analysis suggests that the testing data set has a higher sparsity than the training data set. Figure 6 is a histogram of collective data in different data sets.

**Figure 7:**
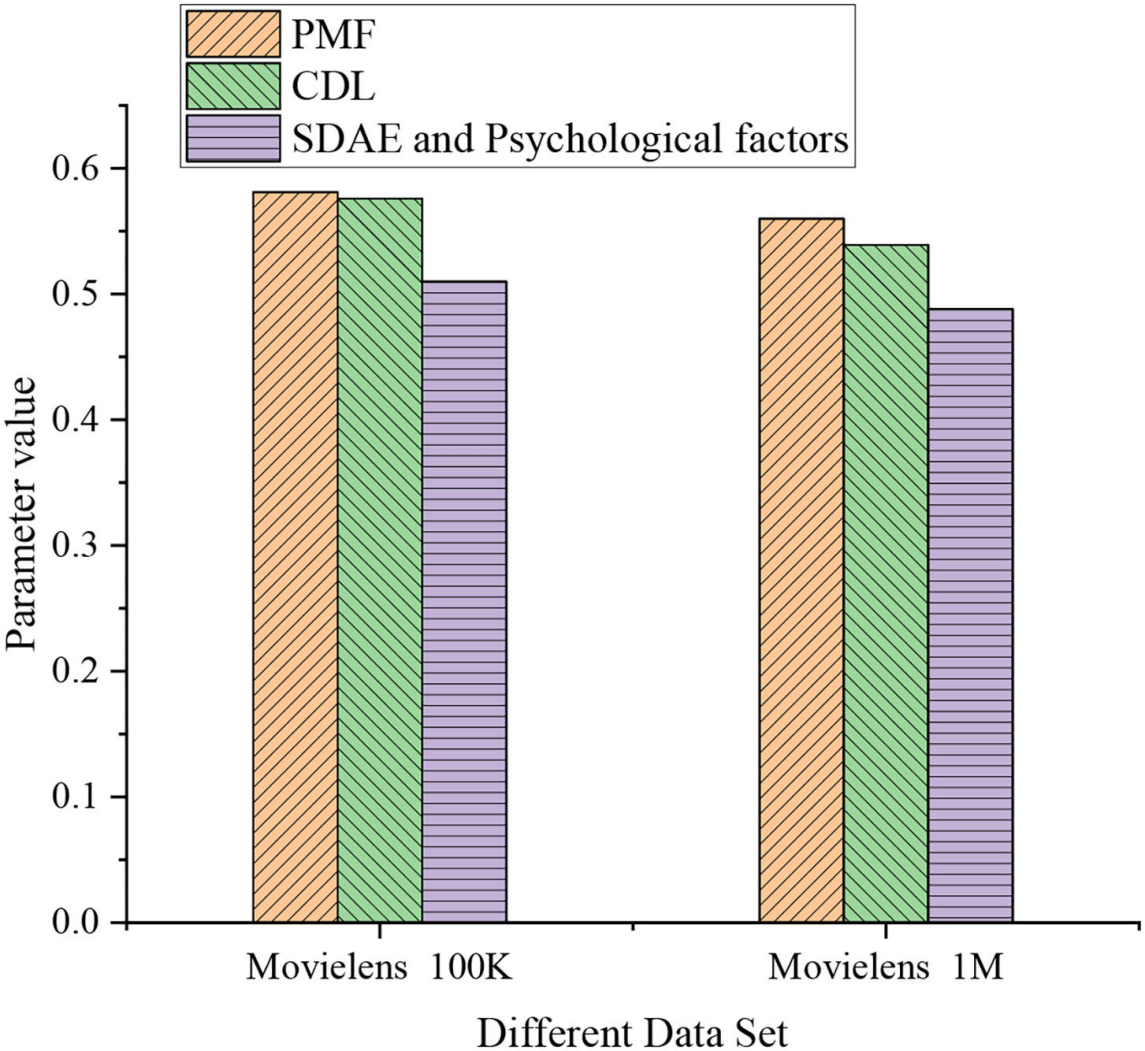
Specific experimental results of different data sets.

Figure 6 provides the experimental results on different data sets. On the training data set, the observed performance parameter of the probabilistic matrix factorization (PMF) model, collaborative DL (CDL), and stacked denoising autoencoder (SDAE) for psychological factors are 0.581, 0.576, and 0.51, respectively. For Movielens 1M data set, the experimental performance parameter of PMF, CDL, and SDAE for psychological factors is 0.56, 0.539, and 0.488, respectively. The data trend indicate that the experimental performance parameters of PMF are higher than those of the other two methods, and parameters on the testing data set are higher than the training data set. Figure 7 demonstrates the specific experimental results of different data sets.

### Analysis of Film EDU-UTL Based on the DL Model

**Figure 8:**
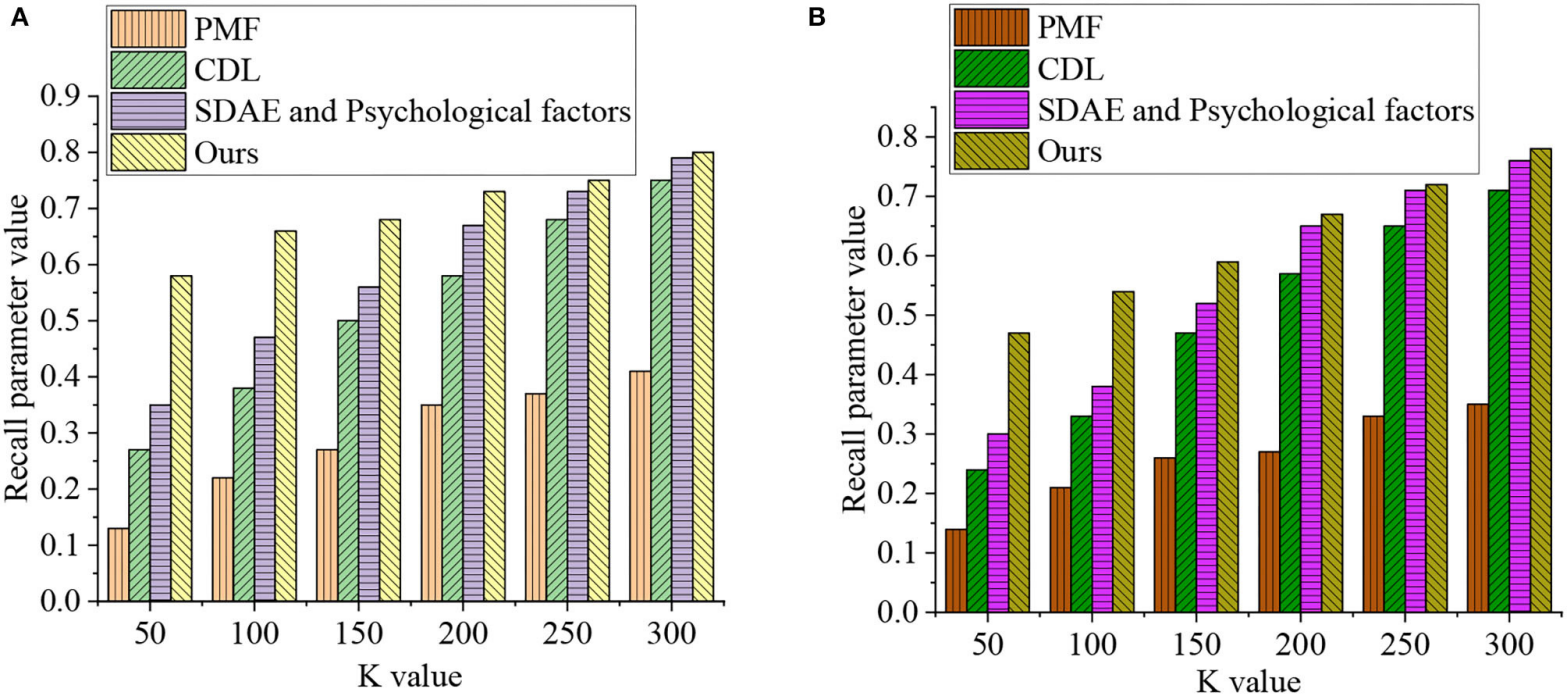
Experimental results of recall and K values on different data sets [**(A)** Movielens 100k training data set and **(B)** Movielens 1M testing data set].

Figure 8 reveals the EDU-UTL analysis results of national films based on the DL model. Figure 8A lists the experimental results on the training data set. When *K* = 50, the psychological factors' Recall of PMF, CDL, SDAE, and the CNN-based deep Q-Learning algorithm is 0.13, 0.27, 0.35, and 0.58, respectively. When *K* = 100, the psychological factors' Recall of PMF, CDL, SDAE, and CNN-based deep Q-Learning algorithm is 0.22, 0.38, 0.47, and 0.66, respectively. When *K* = 150, the psychological factors' Recall of PMF, CDL, SDAE, and the CNN-based deep Q-Learning algorithm is 0.27, 0.5, 0.56, and 0.68, respectively. By comparison, when *K* = 200, the psychological factors' Recall of PMF, CDL, SDAE, and the CNN-based deep Q-Learning algorithm is 0.35, 0.58, 0.67, and 0.73, respectively. When *K* = 250, the psychological factors' Recall of PMF, CDL, SDAE, and the CNN-based deep Q-Learning algorithm is 0.37, 0.68, 0.73, and 0.75, respectively. Lastly, when *K* = 300, the psychological factors' Recall of PMF, CDL, SDAE, and the CNN-based deep Q-Learning algorithm is 0.41, 0.75, 0.79, and 0.8, respectively.

Second, Figure 8B details the experimental results on Movielens 1M testing data set. When *K* = 50, the psychological factors' Recall of PMF, CDL, SDAE, and the CNN-based deep Q-Learning algorithm is 0.14, 0.24, 0.3, and 0.47, respectively. By comparison, when *K* = 100, the psychological factors' Recall of PMF, CDL, SDAE, and the CNN-based deep Q-Learning algorithm is 0.21, 0.33, 0.38, and 0.54, respectively. When *K* = 150, the psychological factors' Recall of PMF, CDL, SDAE, and the CNN-based deep Q-Learning algorithm is 0.26, 0.47, 0.52, and 0.59, respectively. Meanwhile, when *K* = 200, the psychological factors' Recall of PMF, CDL, SDAE, and the CNN-based deep Q-Learning algorithm is 0.27, 0.57, 0.65, and 0.67, respectively. When *K* = 250, the psychological factors' Recall of PMF, CDL, SDAE, and the CNN-based deep Q-Learning algorithm is 0.33, 0.65, 0.71, and 0.72, respectively. Lastly, when *K* = 300, the psychological factors' Recall of PMF, CDL, SDAE, and the CNN-based deep Q-Learning algorithm is 0.35, 0.71, 0.76, and 0.78, respectively. Figure 8 provides the specific experimental results of Recall and *K* values on different data sets.

**Figure 9:**
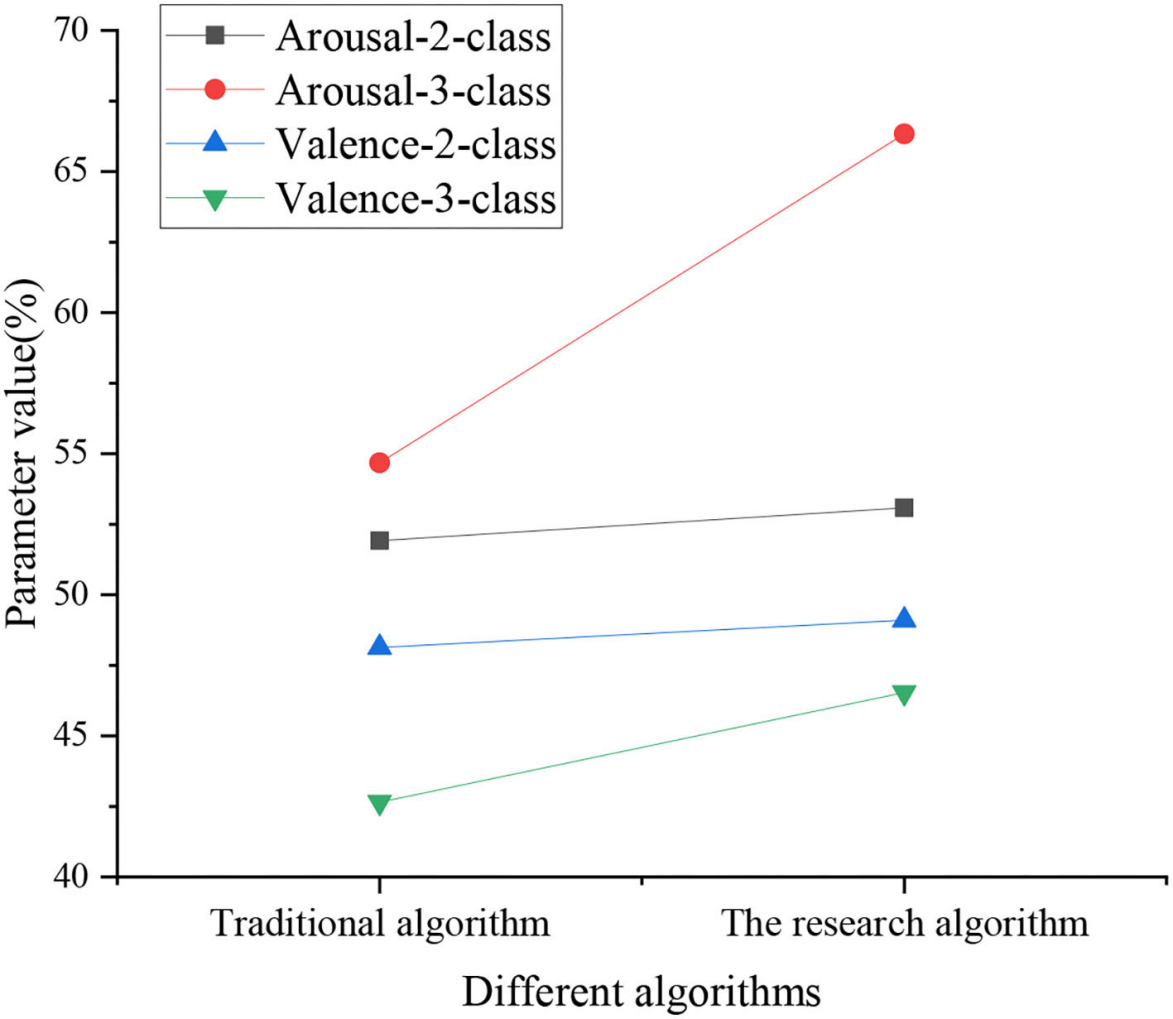
Specific experimental results of different algorithms.

This experiment selects Valence and Arousal as psychological indexes to evaluate the affective states of respondents according to Positive Psychology. Figure 9 reveals that, under the traditional algorithm, the detection accuracy of Arousal-2-class, Arousal-3-class, Valence-2-class, and Valence-3-class is 51.92, 54.68, 48.13, and 42.65%, respectively; for the CNN-based deep Q-Learning algorithm, the detection accuracy of Arousal-2-class, Arousal-3-class, Valence-2-class, and Valence-3-class is 53.08, 66.33, 49.1, and 46.54%, respectively. The data analysis signifies that the detection accuracy of the CNN-based deep Q-Learning algorithm is significantly better than the traditional algorithm. Figure 9 shows the specific experimental results of different algorithms.

**Figure 10:**
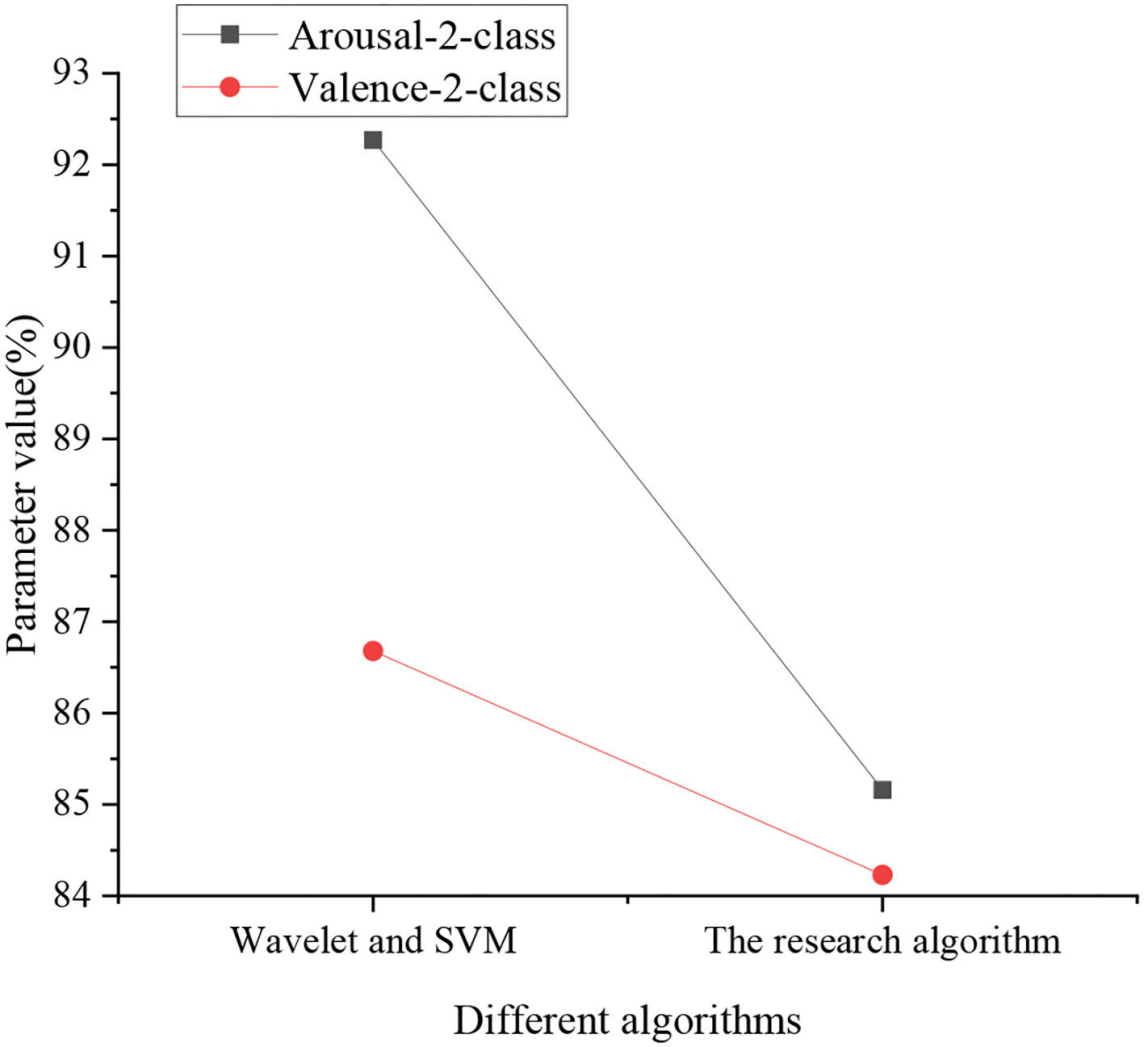
Comparison of Wavelet and SVM with the CNN-based deep Q-Learning algorithm.

Figure 10 specifies the experimental results of Wavelet, Support Vector Machine (SVM), and the CNN-based deep Q-Learning algorithm. Under Wavelet and SVM, Arousal-2-class and Valence-2-class parameters are 92.27% and 86.68%, respectively. Under the proposed algorithm, Arousal-2-class and Valence-2-class values are 85.16 and 84.23%, respectively.

## Discussion

This paper builds up the theoretical basis of the EDU-UTL of national films by consulting the literature. Then, the EDU-UTL of national films is analyzed from the perspective of Positive Psychology by constructing the Q-Learning psychological model and the training model of separable DCNN based on DL. The experimental results prove that the performance parameter and the detection accuracy of psychological factors of the CNN-based deep Q-Learning algorithm are 0.576 and 0.51, respectively. The Recall and *K* value can meet the established requirements by verifying the algorithm on the experimental data set. The main contribution of the research is that the DL model based on Wavelet and SVM recommendation algorithms can effectively enhance the EDU-UTL of national films and improve the efficiency of film education.

## Conclusion

With the rapid development of technology, the world is in an era of rapid transformation. In addition, people in such a social environment show a high comprehensive quality and a strong desire for spiritual pursuit. Especially in recent decades, the film and TV industry has developed rapidly. In this case, the EDU-UTL of film and TV works is becoming more prominent. As the media of the new century, films play a vital role in people's life. This paper focuses on the powerful EDU-UTL behind national films and improves the educational levels through the excavation of EDU-UTL. Movies show many aspects of human civilization from different angles. They can dramatically influence people's psychological feelings, making them realize the importance of peace, prosperity, hard work, determination, and indomitable spirit. Accordingly, this paper investigates the EDU-UTL of national films from the perspective of Positive Psychology by combining the DL method with the Q-Learning psychological model. The results show that when *K* = 300, the Recall of PMF, CDL, SDAE, and the CNN-based deep Q-Learning algorithm reported here is 0.35, 0.71, 0.76, and 0.78, respectively.

To sum up, the CNN-based deep Q-Learning psychological model can improve the EDU-UTL of national films from the perspective of Positive Psychology. Still, the research process has limitations due to the limited research time. The most significant research deficiency is that there are many theoretical results while lacking sufficient experiments to verify the model proposed here, which may affect the universality of the research conclusion. Future research will explore the EDU-UTL of national films based on Big Data Mining technology and improve the precision and accuracy of algorithm recognition through data analysis.

## Data Availability Statement

The raw data supporting the conclusions of this article will be made available by the authors, without undue reservation.

## Ethics Statement

The studies involving human participants were reviewed and approved by Southwest Minzu University Ethics Committee. The patients/participants provided their written informed consent to participate in this study. Written informed consent was obtained from the individual(s) for the publication of any potentially identifiable images or data included in this article.

## Author Contributions

All authors listed have made a substantial, direct, and intellectual contribution to the work and approved it for publication.

## Funding

This study was supported by Youth Innovation Team Development Plan in Shandong Province (2019) and Graduate Education Quality Improvement Plan in Shandong Province (SDYAL19210).

## Conflict of Interest

The authors declare that the research was conducted in the absence of any commercial or financial relationships that could be construed as a potential conflict of interest.

## Publisher's Note

All claims expressed in this article are solely those of the authors and do not necessarily represent those of their affiliated organizations, or those of the publisher, the editors and the reviewers. Any product that may be evaluated in this article, or claim that may be made by its manufacturer, is not guaranteed or endorsed by the publisher.
